# Evening up with the American College of Surgeons

**Published:** 1915-02

**Authors:** 


**Evening Up With the American College of Surgeons.**

We have already deplored the prejudice expressed in some journals toward this organization and, on the other hand, we have felt free to express an opinion, favoring or disfavoring any particular action though, with minor exceptions, we are glad to say that our comments have been sincerely favorable. We feel that it is worth while to say a few words as to the proposal, coming from many sources, to organize a medical body of the same general nature, for the sake of obtaining the same glory and similar initials.

As a matter of fact, there have been for many years, a considerable number of organizations, more or less definitely nonsurgical and more or less specialized, in which membership has been carefully guarded. Whatever eclat may be derived from membership in such organizations has been enjoyed by internists quite as long as by surgeons, much longer than by the American College of Surgeons. Most of these organizations have selected their members with even greater deliberateness though not necessarily with greater care, than has the latter. So far as we can judge, the notion that the College has attempted to make capital for its members by a policy of exclusiveness is justified neither by the authoritative expressions of its officers nor by its roll of members nor even by bare statistics. It has adopted a schedule of requirements that seem perfectly reasonable and it has already admitted about 50% of all the men in the country who can be defined as surgeons in the strict sense, according to any conceivable calculation of economic possibilities. Undue haste in admissions cannot be advocated and there are other organizations which not only include far lower percentages of corresponding specialists but which have boasted of excluding many more applicants than have been received. Announcement to the profession of membership in an organization which implies special honor by its limitations of field and right of exclusion of men even in its field, has long been practiced and without serious objection, though such practice has frequently been criticised.

Considering the numerous societies, special and general, some restricted to internists, some implying such restriction as a common practice, some conferring quite as much honor by limitation according to personal qualification rather than by devotion to this or that general kind of therapeutics, it seems to us that any attempt to "even up" with the surgeons by imitating their organization would be deplorable. We have objected to the use of initials by members of the American College of Surgeons as an encroachment upon the domain of strictly educational institutions and as an imitation of an English method of conferring titles under national conditions which are not closely analogous. Even more strongly, do we object to imitating the College for the sake of giving internists the same privilege. There is ample opportunity, in publishing articles in journals or in making lists of names, for any internist to obtain whatever prestige may attach to membership in any organization. There is no legal or strictly ethical objection, so far as we are aware, to his designating such membership by initials if he prefers, though good taste and good sense favor the use of more understandable designations. The announcement of such connections on office cards and the like, for the purpose of impressing the laity has been objected to, on several occasions and rightly. We feel sure that if any Fellow of the American College of Surgeons made a similar announcement, the College would object quite as strenuously..

There are too many professional organizations, more or less duplicating one another's purposes and interfering with the success of properly concentrated and unified efforts. At the preliminary announcement of the proposed College of Surgeons, we were rather inclined to think that it was of this nature but the practical success of the organization and, especially its phenomenally rapid and large growth has demonstrated that there was a real demand for such an organization, to include in a broader association, various components and, probably ultimately, to supplant previous organizations of less general scope.

If spontaneously, a sentiment should develop toward combining existing societies of internists of different lines of interest or of modifying certain existing organizations to conform to the same model, well and good. But to establish a College of Internists, merely to "even up" would be an imitation that would neither be sincere nor flattering either to imitated or imitators. To establish such a college merely for the sake of adding four letters to one's name would be so puerile that we would even anticipate that greater puerility of striving for a title that would enable medical men to use five or six initials.

While discussing this proposal to secure for internists, a means of obtaining prestige, let us not overlook general principles of real importance. The American College of Surgeons has tried to impress upon its critics, the sincerity of its motives and to make it plain that its object was scientific, educational and humanitarian. If its critics cannot look at the matter in this light, let them at least recognize the fact that no organization, formed for the sake of obtaining prestige, can accomplish that unworthy object, and forbear from attempting to establish a rival organization under circumstances that would preclude any other construction of motives, unless by the tedious process of living down an unfortunate reputation. And let us not forget that, while special and exclusive organizations have their proper place, the organization of any profession that has the highest usefulness, is one in which special lines of interest converge to a focus of common knowledge and efficiency, in which every member is united to every other on a broadly democratic and fraternal basis.

